# Surgical Club of South West England, Spring Meeting 1982

**Published:** 1982

**Authors:** 


### Surgical Club of South West England Spring Meeting – Taunton

### GRUNTZIG DILATATION OF THE ILIAC AND FEMORAL ARTERIES

**W. B. Campbell**
**Bristol Royal Infirmary**

Percutaneous transluminal angioplasty (PTA) using the Gruntzig balloon catheter was undertaken to improve the arterial blood supply of 21 ischaemic lower limbs – a total of 18 iliac and six superficial femoral dilatations. Results have been assessed at 2–23 months (median nine months), as relief of ischaemic pain and improvement in systolic pressures and Doppler waveforms. The patency of dilated and recanalised femoral arterial segments was verified by ultrasonic imaging.

Seventeen of 21 limbs were symptomatically improved following PTA; two were unchanged, and two became worse. Three initial successes reverted to the pre-treatment state during follow-up. Symptomatic improvement did not invariably correlate with ankle pressures, and immediately after dilatation Laplace transform waveform analysis provided a better index of long term success than pressure measurements.

Two iliac arteries became occluded after PTA, and one distal embolus required embolectomy, with a subsequent good result. Minor complications included groin haematomas (3), extravasation of contrast (1), and one embolus not requiring surgical treatment.

These early results are encouraging in the majority of cases treated, but emphasize the need for close collaboration between radiologists and surgeons so that complications can be dealt with promptly.

### CYTOPLASMIC OESTROGEN RECEPTORS IN A DISTRICT GENERAL HOSPITAL

**P. D. Dawkins, G. R. Spencer, A. C. Akehurst**
**Musgrove Park Hospital, Taunton**

It is generally held that Oestrogen Receptor (ER) positive tumours are found in 60–70% of patients with breast cancer. These patients have a lower recurrence rate and a better prognosis than ER negative patients. If recurrence occurs, these patients are also more likely to respond to additive and ablative Endocrine Therapy. There are a number of different approaches possible to estimate the ER content of breast tumours. Techniques involving the use of radioactive tracers, immunological reagents or cytochemical procedures have been proposed. In this hospital in 1979, we set up an ER assay using tritiated oestradiol and the dextran-coated charcoal method, to establish whether it would be possible to provide a routine service in a District General Hospital.

At surgery, part of the resected breast tumour was immediately transported on ice-cold TRIS to the laboratory where it was stored in liquid nitrogen to await assay. There was no evidence that any of the samples deteriorated during storage. Samples were assayed in duplicate, in batches of six using the method described by Johnson et al. A tumour was defined as being ER positive if the result was greater than 8 fmol/mg cytosol protein. 42/61 (69%) were found to be ER positive – a result which is consistent with data from other centres. No correlation between ER status and tumour size, tumour position, axillary node status, blood group, serum CEA and plasma SHBC concentration was demonstrated. The 24 h urinary output of androsterone correlated with ER status ($p < 0.5$). 84 tumours were independently graded by a histopathologist and a correlation between tumour grade and ER content was found. 21/26 (81%) of grade I tumours compared with 11/23 (48%) of grade III tumours were ER positive ($p < 0.05$).

The results of 60 patients who had been followed up for a minimum of 23 months (range 23–36 months) were also presented. 16/41 (39%) ER positive patients had developed recurrence at a mean time of 19 months and five (12%) had since died. In contrast, in the ER negative group 9/19 (47%) had developed recurrence at a mean time of 12 months and six (32%) had since died. Although these figures are not statistically significant because of the small number of patients involved, our results are similar to those found by others.

In conclusion, we found that it was possible to establish an ER assay service in a District General Hospital. However, to obtain material for the assay in good condition, it was essential that there were good channels of communication between the surgeon and the theatre and laboratory personnel.

### BEGINNERS GUIDE TO BREAST RECONSTRUCTION

A. C. Akehurst
Musgrove Park Hospital, Taunton

A series of twenty cases over the last two years was presented with four failures, one due to infection and three due to skin loss. Fifteen were immediate reconstructions, and five were are various intervals post mastectomy.

A decision had been taken to carry out reconstruction in selected cases, in an attempt to reduce the emotional stress of mutilation and the constant reminder to patients of the risk of recurrent malignant disease. The practical problems experienced by patients wearing external prostheses were pointed out.

Selection was important, especially considering the difficulties with large obese patients. Younger women most often ask for implants, but some older ones (especially if slim) are also keen. Upper medially placed tumours are the most difficult to deal with, and the nipple is only preserved if the tumour is not close to it.

A pre-operative positive cytological diagnosis by fine needle aspiration has recently made operation through a laterial incision easier to plan.

Most had sub-cutaneous implants. A few of the smaller implants were placed sub-pectorally, and of these cases one patient had some skin loss but the implant was not interfered with.

The implant size was estimated pre-operatively, from a chart based on bra size and the corresponding volume of external protheses, and was adjusted by measurement of the volume of excised tissue by displacement.

The technique of operation was described using MacIndoe's scissors and illumination via a top light through the skin. It had been recently improved by the development of an arm-rest for the assistant, and the use of a "fish slice" lung retractor to elevate the skin.

### HEROIC SURGERY – MULTIPLE PERFORATION OF COLON DUE TO INGESTED RUBBER BANDS

D. A. Griffiths and J. J. N. Masani
Yeovil District Hospital, Yeovil

Heroic Surgery can be divided into: surgery of the very severely ill patients; surgery of the terminally ill with symptoms that will respond to surgical correction and surgery of dubious value to both surgeon and patient.

Of approximately 700 cases passing through one general surgical firm at Yeovil District Hospital only 20 could be described as heroic.

Surgery of the terminally ill can be of use in the palliation of symptoms, for example, obstructive jaundice, urinary fistulae and bowel obstruction. Good symptomatic relief can be produced by the correct use of surgery in these cases.

Unusual and epic cases of surgery were described and credit given to the anaesthetic, nursing and other staff associated with major, severely ill surgical patients.

An unusual case of large bowel obstruction and perforation due to the ingestion of over 800 rubber bands was described. The patient, an obese woman in her late 50's, presented with extreme shock and a silent distended abdomen and a previous history of thyroidectomy for thyrotoxicosis. At laparotomy an unusual elasticity of the transverse, descending and sigmoid colon was noted with multiple perforations produced by rubber bands. After peritoneal toilet, the patient underwent a sub-total colectomy with primary anastomosis. The patient made an uneventful recovery and is now receiving thyroid replacement for her myxoedema madness. The resected colon was opened and over 800 rubber bands were removed. A search of the world literature has failed to find a similar case.

### TREATMENT OF OBESITY PATIENTS – USING INTERMAXILLARY FIXATION

L. Oldham, Dept. of Oral Surgery,
Musgrove Park Hospital, Taunton

The importance of pre-treatment work-up was stressed. On the first out-patient attendance an estimate was made of the patient's desirable weight – calculated from the Metropolitan Life Insurance tables (1960). This estimated weight gave the clinician, and later the patient, a focus point.

Consultation in a non-clinical environment regarding any previous attempts at weight loss is undertaken. Reasons for failure are discussed, and a complete "talk out" should provide an opportunity to evaluate the patient's personality. An explanation is then given as to what is involved in intermaxillary fixation by the use of cast metal cap splints. Free discussion follows on the difficulty of wearing splints and the possibility of vomiting during fixation, the length of time of fixation, cleaning, talking, and swallowing.

The Dietitian is involved early with a separate interview with the patient away from the clinical area. The Dietitian then reports to the Consultant and the resulting information is fed into the treatment plan. This provides an independent assessment of the patient. A female Dietitian for a female patient is always helpful. A Psychiatrist is involved when the medical background makes this appropriate. A second appointment is then made in the Department of Oral Surgery at which time the radiographs, impressions for splints, and a review of the first appointment notes is made. A date is then fixed for treatment and any haematological tests, blood profiles and biochemistry are obtained. On the third appointment the patient attends as an out-patient at 10.00am and the metal splints are cemented in place. Following this the patient is returned to the ward for an hour. The Oral Hygienist then cleans the splints and the Surgeon checks the occulsion. Analgesics are given as necessary. Fixation wires are placed that afternoon. This avoids the feeling of rushed treatment. The patient is kept in hospital overnight and the wires are adjusted the following morning. If necessary the splints are eased into occlusion. A check is maintained on sleeping and feeding, and the patient is only discharged when confident on these points.

**Follow Up**

The patient is seen at weekly or two weekly intervals for checking of the intermaxillary fixation and the use of red wax over any rough areas of wire. The Dietitian is involved on each visit. The splints are retained until the weight reaches the desired weight, or the patient requests the splints to be removed. After this introduction Mr. Oldham presented four cases: two who had failed in treatment and two who had been successful. Accurate weight recording graphs during treatment, photographs of the patients' faces, and behavioural studies all combined to help the treatment of patients with obesity problems.

In summary it was felt that treatment of obesity by using intermaxillary fixation did have a part to play in the control of obese patients, who were either unsuitable for surgical techniques or as a pre-operative measure to render the patients more suitable for surgery. Close co-operation with the Surgeon is mandatory as with other disciplines.

### SURGICAL TREATMENT OF MORBID OBESITY

**C. D. Collins**
**Musgrove Park Hospital, Taunton**

Twelve patients of more than twice the normal body weight have been treated surgically over the past seven years. The operation was the classical Payne and de Winde jejuno-ileal bypass, anastamosing the end of jejunum divided 14 inches from the duodenojejunal junction to the side of the terminal ileum four inches from the ileo-caecal valve. There were no serious complications or deaths. Of the twelve, one was a man whose weight reduced from 25 stone to 17 stone at which level the weight was maintained for over 18 months. For various, mostly psychological reasons he requested a reversal which was carried out, his weight subsequently returning to 23 stone. The eleven females did well losing an average 35% of their pretreatment weight in 18 months and maintaining the weight loss for up to 5½ years. The costs of hospital care both in and outpatient, including medications, during the first year were calculated and considered to be not excessive. The medical time involved in pre and post-operative care was, however, considerable. Attempts at achieving weight reduction by jaw splinting were also described and although tolerated by a few patients for several months, the effects were considered so short-lived as to regard this technique primarily as a useful antecedent to intestinal bypass surgery rather than a definitive treatment.

### ABDOMINAL DRAINAGE POST CHOLECYSTECTOMY

**T. T. McCormack, P. D. Abel, C. D. Collins**
**Musgrove Park Hospital, Taunton**

A prospective trial of drainage following cholecystectomy was carried out. Three types of drain were used, (I) Redivac Drain; a suction drain with a high negative pressure (maximum 440 mm. Hg.), (II) Drevac Drain; a suction drain with a low negative pressure (maximum 155 mm. Hg.), (III) Robinson Drain; a static drain with no significant negative pressure

120 patients undergoing cholecystectomy between April 1981 and February 1982 were included. 84 patients (70%) underwent cholecystectomy alone and 36 patients (30%) had exploration of the common bile duct. Choice of drain was randomly made from sealed envelopes. Drainage fluid was sent for culture from each container at 48 hours. Drains were removed on the morning of the 3rd post operative day.

There were 40 patients in each group. The volume of drainage from the Drevac drain was significantly less than from the other two drains during the first and second 24 hours. Organisms were less frequently cultured from the fluid in the Drevac container (3.2%) than Robinson (10.3%) or Redivac (11.1%). There was no significant difference in rates of wound infection (mean 2.5%), chest infection (mean 3.3%) or hospital stay (mean 10 days).

This trial did not demonstrate any clinically useful difference between high pressure, low pressure or static drainage following cholecystectomy.

### PROSTATITIS – THE TAUNTON EXPERIENCE

P. J. O'Boyle
Musgrove Park Hospital, Taunton

Prostatitis is an emotive and emotional subject. The diagnosis is made by exclusion of bacterial infection, particularly in the expressed prostatic secretion. Anaerobic, chlamydial and herpes simplex infection should be remembered. A three-year review of prostatitis patients demonstrated a classical prostatitis personality, and emphasised the importance of the volume frequency voiding chart, both as a method of investigation and of treatment. These patients undoubtedly merit full investigation to exclude more sinister pathology.

Treatment is notoriously unsatisfactory despite the wide chemotherapeutic armamentarium. The place of surgery is likewise limited but can be spectacularly successful. The most frequently successful treatment modalities employed in Taunton were the volume frequency voiding chart, Phenoxybenzamine, and Flurbiprofen.

Finally, there can be no doubt that these patients are extremely demanding in terms of time. A sympathetic ear may be of paramount importance in helping the unfortunate patient to accept, and come to terms with his illness.

### GUTTER TREATMENT FOR INGROWING TOE-NAILS

D. J. Kerle
Musgrove Park Hospital, Taunton

The treatment of ingrowing toe-nails (IGTs) has a twofold objective – relief of pain and prevention of recurrence. Recurrence rates as high as 80% are reported when treated by junior surgical staff.

Gutter treatment for IGTs depends upon a silastic sleeve insert to protect the inflamed lateral nail fold from the jagged nail margin. The silastic gutter and nail are cut together as the nail grows. Advantages of this method are numerous. Criticism has been directed at

(1) Cost of the commercial toe-nail kit
(2) Limited improvement of recurrence rates.

These criticisms have been investigated in Taunton. A cost reduction of 90% was achieved by using cut lengths of nasopharyngeal suction cannulae. Our technique employed the Lockhart-Mummery fistula probe. It's cross sectional C shape makes it an ideal tool for gutter insertion. The plastic cannula is mounted on the probe, incised lengthwise, advanced over the offending nail margin and lodged under the epinychium. Local anaesthetic is used. Suture fixation is occasionally necessary.

The results of 14 gutters are presented. Six are reviewed at six months. To date there have been no recurrences. manual workers and bilateral IGTs are prone to gutter dislodgement.

In the absence of significant subungual pus gutter treatment is recommended as a simple, cheap and effective technique for primary ingrowing toe-nails.

### THE VALUE OF INVESTIGATING PAINLESS HAEMATURIA

A. P. Barlow
Musgrove Park Hospital, Taunton

It is current practice to investigate any patient presenting with painless haematuria with an IVU and endoscopy. A cause for macroscopic haematuria will be found in 90% of cases whilst for microscopic haematuria a diagnosis will be made in 80% of cases. Although the cause is often benign a malignant condition will be found in 20% and 10% of patients respectively.

In Taunton between 1970–78 114 patients were investigated for painless haematuria with no diagnosis resulting. 34% were discharged without follow up whilst the majority of the remainder were only seen once. However, 12 of the patients, because of further haematuria, were re-investigated, and nine were subsequently found to have a cause for this, six patients had a renal carcinoma and three patients a bladder tumour.

This experience suggests that even when initial investigations are normal as many as 10% of patients with macroscopic haematuria will have further haematuria and in most of these a life-threatening condition will be demonstrated.

It is suggested that follow up should be for five years and that the IVU and endoscopy, at least, must be repeated if haematuria is again detected.

### BLOOD GROUP ANTIGENS AND BLADDER CANCER

**P. D. Abel and P. J. O'Boyle**
**Musgrove Park Hospital, Taunton**
**Ten Feizi, Susan Thorpe, G. Slavin**
**Clinical Research Centre, Harrow**

It will be important to establish whether the deletion of blood group (ABH) antigens on bladder tumour cells is of value as a prognostic indicator in determining whether local invasion and metastases are likely to occur.

In a preliminary study to determine blood group antigen status on the normal transitional cell epithelium, formalin-fixed paraffin-embedded sections were compared with cryostat sections. Specific anti-blood group antigen sera was, used with indirect immunoflourescence studies to demonstrate the distribution of the blood group A and H antigens.

Results showed that cryostat prepared sections were superior to formalin-fixed paraffin-embedded sections in preserving antigens for detection. Distribution of antigen in cryostat sections was seen to be even throughout the mucosa in contrast to the patchy distribution seen in formalin-fixed paraffin-embedded sections. This suggests that blood group antigens were being extracted during the progressing of the formalin-fixed paraffin-embedded sections.

Previous work to detect blood group antigens in normal and neoplastic transitional cells have mainly used formalin-fixed paraffin-embedded sections and thus their accuracy must now be in doubt.

Further studies are in progress to compare cryostat and formalin-fixed sections using malignant material.

### FINE NEEDLE ASPIRATION CYTOLOGY OF LUMPS IN THE BREAST

**D. R. Turner**
**Musgrove Park Hospital, Taunton**

The pathologist normally makes a histological diagnosis using the relationships between individual cells and the adjacent connective tissues. Cytological features of cells may also be considered. In fine needle aspiration cytology the cell relationships are largely destroyed and much greater demands are made upon the cytological features of individual cells. The resultant diagnosis must inevitably be less secure. One is left with a dilemma whether or not to use a technique which is more convenient for the patient but with some loss of confidence in the diagnosis achieved.

Fine needle aspirate cytology became popular in Sweden in the 1960's culminating in the publication of a large series of 3500 cases from the Karolinska Institute. There were 843 +ves using fine needle aspiration cytology and only one false positive. There was a however a 30% false negative rate. Therefore a negative result should be regarded as **no** result.

Webb in 1955 suggested that if the operator and the cytologist were one and the same person the results could be improved. Presumably this is at least in part due to using the clinical impression of the lump as a guide to the cytological diagnosis.

The problems of interpretation include:—

(1) Florid reactive changes in benign lesions which can simulate malignancy.
(2) Cells from a well-differentiated carcinoma can look cytologically very normal.
(3) A poorly differentiated tumour may be obviously malignant but cytology may give little clue as to its type.
(4) The extent of experience of the pathologist.

### A COLLABORATIVE TRIAL TO EVALUATE THE NEED FOR MASTECTOMY IN THE MANAGEMENT OF EARLY BREAST CANCER

**C. Teasdale**
**Bristol Royal Infirmary**

A prospective randomized controlled trial has been designed in clinical Stage I and II invasive breast cancer to determine whether local tumour excision, with breast conservation, plus radical radiotherapy gives results comparable to simple mastectomy plus radical radiotherapy, in terms of recurrence and survival. Cosmetic results and psychological sequelae will also be studied. Funding has been provided by the Cancer Research Campaign and the trial is scheduled to begin in Autumn 1982.

**Trial Designs**

Suspected breast cancer
Clinical Stage I or II
↓
Cytological and/or histological
confirmation of tumour any of these may be employed. } — { Needle biopsy

Drill biopsy

Fine needle aspiration

Frozen section

↓

*Continued on page 36*

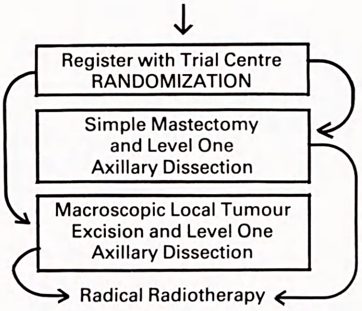

The trial will be administered and co-ordinated from two centres – the C.R.C. Trials Centre at Kings College Hospital (central randomization, data collection and analysis office) and Bristol Royal Infirmary. The aim is to enter 2000 analysable patients in 3–4 years so that the chance of demonstrating no significant difference between the groups (<7%) with confidence will be 95%. The approval of local ethical committees for the study is advised and Surgeons and Radiotherapists in the South West of England will be encouraged to enter their patients.

### "SURGICAL FOLLOW-UP – IS IT WORTHWHILE?"

**J. A. B. Collier, T. McCormack, P. Abel, S. Hayes, C. D. Collins**

This presents the preliminary findings of the Taunton Outpatients Follow-up Survey (first 50 cases). Outpatient doctors and patients completed a questionnaire at the patient's first appointment after surgery. A postal questionnaire was then later sent to these patients and their General Practitioners. The aim was to assess the views of all three on the worthiness of routine post-operative surgical follow-up appointments.

### Results

Hospital doctors felt patients would be as well followed up by general practitioners in 76% of cases and that General Practitioners had sufficient knowledge to see 92%. General Practitioners felt that 84% cases need not have attended Outpatients and expressed willingness to follow up 96%. Hospital doctors felt District Nurses capable of seeing only six cases, whereas General Practitioners felt them capable of seeing all but six. 86% of patients had already seen their District Nurse.

Only eight patients had not seen their General Practitioner prior to the outpatient appointment, 27 had seen him for certificates, 28 for advice, 10 for medicines, 12 for other health problems.

Only four patients were given a second outpatient appointment, only nine had their management changed. Less than half saw doctors previously seen in hospital. One-third took time off work to attend outpatients.

### Conclusion

For maximal utilisation of resources routine post-operative outpatient follow-up is unnecessary – General Practitioners being willing, and judged sufficiently knowledgable to do this. Against this 80% of patients preferred to attend outpatients and surgeons like to see the results of their labours.

### DISSOLUTION OF GALL STONES

**S. Hayes and A. C. Akehurst**

A clinical study was performed over the past 24 months at Musgrove Park Hospital on the eficacy of Chenodeoxycholic Acid (CDCA) in the treatment of gall stones.

Number of patients: 27. Ages ranged from 25 to 82 years. Sex ratio 3m:24f.

Medical therapy was assigned in cases thought unfit for surgery, patients with favourable criteria and at the request of individual patients.

A standard regime as recommended by the literature was used. This included:

(1) Low fat diet (=40 gm/day)
(2) CDCA 10–15 mg./kg (nocte)
(3) Check X-ray at six-monthly intervals

Ultrasound examination was performed occasionally but was thought not to be as reliable as oral cholecystogram in our experience.

CDCA was chosen because of its "safety record", its action as a choleretic and as an inhibitor of HMGCoA Reductase, an enzyme which controls the rate of cholesterol synthesis in bile. This enzyme is increased in patients with lithogenic bile. Attention was given to proper dosage and administration in accordance with other recent studies and the manufacturers directions.

Results were in accordance with previous published figures – our rate of total dissolution was 32% of stones dissolved in the presence of a functioning gall bladder and sized 1 cm. and radiolucent. There were five cases of diarrhoea but only two patients withdrew because of this. Abnormal LFTs were noted only in patients with CBD stones.

Maintenance therapy was not initiated. Recurrence has not been evaluated.

